# Photoprotective Effects of Phytochemicals on Blue Light-Induced Retinal Damage: Current Evidence and Future Perspectives

**DOI:** 10.3390/nu17020331

**Published:** 2025-01-17

**Authors:** Wan-Ju Yeh, Cin Yan, Chi-Hao Wu

**Affiliations:** Graduate Program of Nutrition Science, School of Life Science, National Taiwan Normal University, Taipei 11677, Taiwan; wandayeh@ntnu.edu.tw (W.-J.Y.); michelle88113017@gmail.com (C.Y.)

**Keywords:** blue light, light-emitting diodes, phototoxicity, phytochemical, polyphenol, retina

## Abstract

The widespread use of light-emitting diodes (LEDs) has increased blue light (BL) exposure, raising concerns about its potential adverse effects on ocular health. Prolonged exposure to BL has been implicated in the pathogenesis of various retinal disorders, including age-related macular degeneration (AMD), primarily through mechanisms involving oxidative stress and inflammation mediated by the overproduction of reactive oxygen species (ROS). This review synthesizes current evidence on the photoprotective properties of dietary bioactive compounds, (e.g., anthocyanins, curcumin, quercetin, myricetin, and resveratrol), with a focus on their potential to mitigate BL-induced retinal damage. Accumulating research suggests that dietary antioxidants, particularly polyphenols, may offer photoprotective benefits. These phytochemicals act by neutralizing ROS and enhancing the retina’s endogenous antioxidant capacity. Based on these findings, this review advocates for a food-first approach in future investigations, emphasizing the development of evidence-based dietary recommendations to bolster retinal health and mitigate the risk of BL-related ocular diseases. Considering the current lack of empirical clinical studies examining the impact of BL on human ocular health, future research in the field of BL hazard should prioritize two key approaches: conducting large-scale epidemiological dietary surveys and implementing clinical trials on functional ingredients that have demonstrated beneficial effects against photodamage in preclinical animal studies.

## 1. Introduction

Blue light (BL), defined as high-energy visible light with wavelengths ranging from 400 to 500 nm, has become increasingly prevalent in contemporary society due to the widespread use of electronic devices and energy-efficient lighting solutions such as LEDs [1]. Unlike traditional incandescent bulbs, which emit a broader spectrum of light, LEDs are characterized by their high intensity and significant emission of BL [2]. This shift in lighting technology has raised critical concerns about the potential health implications of prolonged BL exposure, particularly in relation to visual function and retinal integrity [3].

Excessive BL exposure can penetrate the retina, leading to photochemical damage and contributing to conditions such as age-related macular degeneration (AMD) and other forms of retinopathy [4]. The underlying mechanisms of BL-induced retinal damage are primarily associated with the generation of reactive oxygen species (ROS), such as singlet oxygen, superoxide anion, and hydrogen peroxide, which induce photooxidative stress on retinal tissues [5]. ROS accumulation results in cellular apoptosis and inflammation, compromising retinal function [6]. Photoreceptor cells and the retinal pigment epithelium (RPE), crucial for retinal health, are the primary targets of this oxidative stress [7]. The RPE becomes dysfunctional under heightened oxidative stress, further exacerbating retinal damage [8].

BL exposure induces lipid peroxidation in photoreceptor outer segments (OS), which are rich in polyunsaturated fatty acids, making them highly vulnerable to oxidative damage [9]. Studies in rodent models show that BL exposure leads to retinal damage, such as reduced photoreceptor density in the outer nuclear layer (ONL), nuclear pyknosis, and RPE cell dysfunction [10]. For example, Wistar rats exposed to 2 h of BL (460 nm) demonstrated increased oxidative stress marker 8-hydroxy-2′-deoxyguanosine (8-OHdG) and photoreceptor apoptosis [11]. Additionally, exposure to commercial white LEDs induces photoreceptor apoptosis via caspase-independent pathways [12].

The blood–retinal barrier (BRB) is one of the earliest sites of BL-induced damage. Breakdown of the BRB allows macromolecules and harmful substances to infiltrate the retina, disrupting its microenvironment [13,14]. Furthermore, chronic exposure to cool-white LEDs triggers rhodopsin-Ser334 phosphorylation, contributing to photoreceptor apoptosis [12]. Müller cells and microglia respond to oxidative stress, contributing to retinal inflammation and degradation [15]. These findings underscore the central role of oxidative stress in BL-induced retinal injury and highlight the protective potential of antioxidants such as *N*-acetylcysteine [16]. Therefore, maintaining oxidative balance is crucial for retinal health and preventing photodamage.

Certain antioxidants have been shown to effectively combat photodamage, suggesting that naturally occurring phytochemicals may also possess anti-BL toxicity properties [17,18]. Consumption of polyphenols may help prevent AMD [19]. The age-related eye disease study (AREDS) [20] and AREDS2 [21] trials have both revealed that taking an antioxidant supplement could reduce the risk of developing advanced AMD. A recent review highlights the potential benefits of antioxidants and omega-3 fatty acids in managing AMD, emphasizing the importance of diet and oral supplements. Key compounds such as lutein, zeaxanthin, astaxanthin, and vitamins A, C, and E, along with zinc, are highlighted for their protective effects against oxidative stress and inflammation. The Mediterranean diet also shows promise in reducing AMD risk [22]. Thus, phytogenic products and phytochemicals with a high potential for reducing BL toxicity need further investigation. In this context, dietary bioactive compounds, particularly those with antioxidant properties, have garnered increasing attention for their potential protective effects against BL-induced toxicity. Polyphenols found in various fruits and vegetables have demonstrated significant antioxidant capacities that may effectively counteract the harmful effects of BL [23,24].

This review aims to comprehensively evaluate the current understanding of the protective roles of dietary bioactive compounds against BL toxicity. By highlighting the importance of dietary interventions as a proactive strategy for preserving retinal health, this work underscores the necessity for future research to explore the optimal intake and bioavailability of these compounds. Ultimately, the goal is to establish evidence-based dietary recommendations that enhance ocular health in an increasingly BL-saturated environment, ensuring better visual outcomes for the population at large. Because certain antioxidants effectively combat photodamage, naturally occurring phytochemicals may also possess anti-BL toxicity potential [17,18]. Thus, phytogenic products and phytochemicals with a high potential for reducing BL toxicity need further investigation. In this review, we summarize and discuss the photoprotective properties of dietary bioactive compounds, such as polyphenols and plant extracts, against BL damage in vitro and in vivo, along with the underlying mechanisms.

## 2. Materials and Methods

A comprehensive literature search was conducted in PubMed, Scopus, and Web of Science using keywords such as “blue light”, “flavonoid”, “oxidative stress”, “phytochemicals”, “polyphenol”, and “retina”. Studies published between 1994 and 2024 were considered for inclusion. Only articles written in English and focused on retinal cell culture, animal models, or human studies of retinal damage due to BL exposure were included. Studies examining the protective effects of antioxidants or phytochemicals on retinal health were prioritized.

## 3. Naturally Occurring Phytochemicals and Plant Extracts with Protective Potential Against BL-Induced Retinal Damage

Given the importance of photooxidative stress in retinal pathology, supplementing antioxidants in response to light challenges could be a possible strategy to avoid BL risks [25]. This review highlights current information on using naturally occurring phytochemicals (Table 1) and plant extracts (Table 2) to protect against BL photodamage and explores the possible mechanisms that underlie them in the following section.

### 3.1. Polyphenols

#### 3.1.1. Resveratrol and Resveratrol Analogs

Resveratrol is a polyphenol compound abundant in grape skin. It is known for its antioxidant properties, beneficial biological functions, and low toxicity. In humans, resveratrol can be rapidly absorbed and metabolized into glucuronide and sulfated derivatives, which are eliminated via the urine [42]. Although peak plasma resveratrol levels may reach 25 ng/mL (0.11 μM), this is still below the quantities effective in vitro (>1000 ng/mL or 5–50 μM) [42,43]. The oral bioavailability of resveratrol is <1% [44].

The effects of resveratrol on BL phototoxicity in humans and animals have yet to be elucidated. Resveratrol protects human ARPE-19 [45] and keratinocyte HaCaT cells [46] against oxidative damage caused by ultraviolet (UV) A. Kang et al. [26] demonstrated that resveratrol and its analog piceatannol protected A2E-laden ARPE-19 cells that were exposed to BL at a concentration range of 7.5–60 μM. Treatment with resveratrol and piceatannol reduces oxidized A2E product formation and intracellular A2E accumulation. Among the compounds tested, resveratrol-3, 4′-O-β-d-diglucoside had the strongest photoprotective properties in ARPE-19 cells [26]. A case report of an 82-year-old patient with dry AMD revealed that a combination of nonblue LED light exposure at 590, 660, and 850 nm and resveratrol supplementation for 6 months improved retinal morphology and visual acuity [47]. Combining 100 mg microencapsulated trans-resveratrol with quercetin, ferulic acid, vitamin D3, and inositol hexaphosphate (a copper/iron/calcium chelator) improved choroidal blood flow and RPE function in patients with treatment-resistant macular degeneration [48]. Additionally, intravitreal injection or topical instillation of resveratrol at a dose of 5–50 mg/kg/day suppressed the development of retinal angiogenesis in mice [49].

Resveratrol is a phytoalexin produced by plants in response to environmental injuries including solar radiation or pathogen infection [50]. Resveratrol can be photoactivated using blue LED light and may be used in photodynamic therapy as an antimicrobial photosensitizer [51]. This may result in singlet oxygen photogeneration, raising health and safety concerns for persons who are frequently exposed to high-BL environments while taking high-dose resveratrol supplements. As a result, resveratrol may play a dual role as a photoprotective or phototoxic agent, depending on the action spectrum caused by light exposure.

#### 3.1.2. Curcumin

Curcumin is a natural yellow pigment extracted from the rhizomes of *Curcuma longa* L. In a rodent model of acute photodamage, Wistar rats were exposed to 1000 lux of cool-white light for 3 h. Photoreceptor function and retinal morphology were assessed using ERG and quantitative histology. Mandal et al. [27] showed that dietary supplementation with 0.2% curcumin for 2 weeks prevented the loss of photoreceptor function and the thinning of the ONL from light-induced retinal degeneration. Curcumin also inhibited H_2_O_2_-induced cell death in 661 W photoreceptor and ARPE-19 cell lines by upregulating the expression of HO-1 and thioredoxin. Furthermore, 70% ethanol extracts of *Curcuma longa* L. and its curcuminoid compounds (curcumin, demethoxycurcumin, and bisdemethoxycurcumin) have conferred photoprotection by suppressing proapoptotic c-abl and p53 genes in A2E-laden ARPE-19 cells exposed to BL (420–470 nm; 9.4 mW/cm^2^). However, at concentrations over 20 μM, curcumin is cytotoxic to ARPE-19 cells [28].

Curcumin, which is phytochemically similar to resveratrol, is emerging as a photosensitizer capable of producing ROS when exposed to blue LED light and has been applied in antimicrobial photodynamic therapy [52,53] and food preservation [54]. The working concentrations of curcumin, ranging from 0.5 μM to 6.1 mM, have been effectively used in antimicrobial photodynamic therapy studies [55]. However, curcumin is phototoxic to mammalian cells [56,57]. Curcumin absorption spectra in aqueous buffers show a distinctive broad peak at 430 nm and a small shoulder at 355 nm. When curcumin is converted to its ketone form, the maximum absorption in the UV region occurs at approximately 389 nm [58]. Curcumin has drawbacks due to photodegradability, poor water solubility, and instability at physiological pH, resulting in rapid degradation and low bioavailability [59]. Curcumin conjugates and analogs have been developed to improve water dispersibility, chemical stability, absorption, and overall bioavailability [60]. However, when compared to other natural photosensitizers such as riboflavin, anthraquinones, and porphyrins, this fact may raise ocular safety concerns [55]. Several studies have addressed these questions, but the exact photobiological responses in the retina have yet to be determined.

#### 3.1.3. Anthocyanins

Anthocyanins are water-soluble pigments abundant in red, blue, and purple fruits and vegetables such as berries, plums, and eggplants [61]. According to data from the Phenol-Explorer database between 2013 and 2016, the average dietary flavonoid intake among US adults is approximately 379.1 mg per 1000 kcal/day [62]. Moreover, daily anthocyanin intake is estimated to range from 3 to 43 mg/day among nations [63]. The dietary intakes of the six major anthocyanins, including cyanidin, delphinidin, malvidin, pelargonidin, peonidin, and petunidin, among Australian adults, are estimated to be 9.33, 5.46, 14.01, 0.22, 1.97, and 1.96 mg/day, respectively [63].

The therapeutic potential of anthocyanins in ophthalmology has been demonstrated in in vivo and in vitro studies, including rhodopsin regeneration, smooth muscle relaxation, and blood circulation improvement studies [64]. In pigmented rabbits, oral administration of cyanidin-3-glucoside (50 mg/kg/day) and its metabolite ferulic acid (21.61 mg/kg/day) for 3 weeks ameliorated photoreceptor structural and functional abnormalities during short-term high-intensity light exposure. The activation of the Nrf2/HO-1 pathway and suppression of inflammatory responses are postulated to be protective mechanisms [65]. More than 250 known natural anthocyanins are found in glycoside form because free anthocyanins are unstable. A human 13C-tracer study conducted by Czank et al. [66] showed that the bioavailability of isotopically labeled cyanidin-3-glucoside was 12.38%, and the peaked serum concentration of ferulic acid was low (0.94 μM), 48 h after an intake of 500 mg cyanidin-3-glucoside. In a cellular model [29], cyanidin-3-glucoside at a concentration range of 10–50 μM was found to protect cells against BL damage from scavenging ROS, suppress A2E photooxidation, and prevent GSH reaction with photooxidized-A2E, leading to cell survival.

Nine monoglycosylated anthocyanins were identified in bilberry extracts, including cyanidin, delphinidin, malvidin, and petunidin. Malvidin-3-glucoside (oenin) provides the most potent photoprotection, and all other identified anthocyanins can also increase resistance to A2E-mediated membrane disruption [67]. Yacout and Gaillard [30] reported that, compared with pelargonidin 3-glucoside (callistephin), malvidin-3-glucoside more effectively protected ARPE-19 cells from BL-induced mitochondrial damage and inhibited ROS production. Additionally, treatment with anthocyanin-rich blueberry extracts delays cellular senescence in white LED light-exposed ARPE-19 cells [38] and protects 661 W cells from BL-induced cell death [39].

Grape skin contains a variety of polyphenols, including resveratrol, catechin, epicatechin, anthocyanin, quercetin, and phenolic acid; consequently, it possesses potent antioxidant properties [68]. According to Zhao et al. [40], grape skin extracts prevent BL-induced apoptosis in A2E-laden ARPE-19 cells. As grape skin extracts have no therapeutic action in GRP78 knockdown cell lines, the protective mechanisms are likely associated with the inhibition of the ER stress-mediated intrinsic apoptotic pathway. Yu et al. [69] observed that administering grape or lutein/zeaxanthin-rich marigold extracts prevented lipofuscin accumulation, hydroxynonenal-adduct formation, actin destabilization, and age-related photoreceptor dysfunction in β5^−/−^ mice. These data suggest that a diet rich in polyphenol antioxidants benefits retinal health.

Previous studies have examined anthocyanin levels in eyes. The total anthocyanin concentration in the ocular tissue of pigs fed 1–4% blueberries for 4 weeks was calculated to be 709 pg/g tissue (1.58 pmol/g tissue). Furthermore, of the 11 anthocyanins identified in the eye, malvidin glycosides were the most abundant [70]. In an ocular distribution study with Wistar rats given 100 mg/kg body weight (b.w.) of blackcurrant anthocyanins orally, the maximum concentration in the entire eye reached 115 ng/g tissue. The anthocyanin level in the retina following i.p. injection (500 mg/kg b.w.) was as high as 6.89 μg/g tissue, which was similar to that in plasma [71]. These results demonstrate that natural anthocyanins can cross the BRB and accumulate in the retina, and may act as neuroprotective agents against light damage.

#### 3.1.4. Quercetin, Quercetin-3-*O*-α-l-Arabinopyranoside, and Myricetin

A decade of research has focused on the use of quercetin in ophthalmic diseases, including cataracts, glaucoma, autoimmune Graves’ orbitopathy, keratoconus, retinoblastoma, and various other ocular surface diseases [72]. Systemic administration of quercetin (50 mg/kg b.w.) to rats for 6 d prior to a light insult protected the retinal tissue from photooxidative damage caused by high-intensity white fluorescent light (3000 lux). The levels of 8-OHdG in the RPE and ONL, as well as the number of TUNEL-positive cells in the photoreceptors, were decreased in quercetin-treated rats. Moreover, quercetin restored ERG-based scotopic a- and b-wave losses [31]. At a dose of 50 μM, quercetin protected A2E-laden ARPE-19 cells from BL cytotoxicity. Protective mechanisms are postulated to involve ROS scavenging and diminishing A2E-epoxide formation [29]. Given that reactive dicarbonyl species, including glyoxal and methylglyoxal, can be released during bis-retinoid photodegradation [73], Wang’s study found that quercetin reduced methylglyoxal-derived hydroimidazolone (MG-H1) protein adduct levels and RAGE gene expression in A2E-laden cells exposed to 430 nm BL irradiation [29]. Quercetin also protects ARPE-19 [74] and RGC-5 ganglion cells [75] from H_2_O_2_ oxidative damage and apoptotic cell death. However, in Ccl2/Cx3cr1^−/−^ mice, which spontaneously develop AMD-like retinal lesions, the systemic administration of quercetin (25 mg/kg/day) did not alter the course of retinal lesions [74].

Quercetin-3-*O*-α-l-arabinopyranoside is a plant flavonoid found in *Vaccinium uliginosum* L. extracts and is abundant in berries such as guava. Kim et al. [32] used this flavone in a series of investigations. In vivo experiments revealed that orally administering quercetin-3-*O*-α-l-arabinopyranoside (25–100 mg/kg b.w.) reduced the thinning of the INL, OPL, and ONL in the retina, protecting against short-term high-intensity blue–green light (480 nm)-induced retinal damage in BALB/c mice. In the cellular model, quercetin-3-*O*-α-l-arabinopyranoside inhibited A2E internalization, protecting A2E-laden cells against BL-induced apoptosis.

Myricetin was first isolated from the bark of *Myrica nagi* Thunb. (Myricaceae); it is structurally related to quercetin, kaempferol, morin, and fisetin. Vegetables, berries, apples, oranges, nuts, and tea are the most common dietary sources of myricetin. The estimated daily consumption of myricetin in European and Korean adults is between 0.8 and 4 mg/day [76]. A recent in vitro study by Ortega et al. [77] suggested that myricetin and quercetin upregulate rhodopsin gene expression and maintain the proper folding and stability of rod opsins. HPLC revealed that myricetin and quercetin bind to unliganded opsins, facilitating retinal binding to such opsin proteins. The study demonstrated that giving myricetin and quercetin (0.02–2 mg/kg b.w.) before BL exposure protected the retina from morphologic abnormalities and preserved its function in *Abca4-Rdh8*^−/−^ mice exposed to short-term high-intensity BL (10,000 lux for 45 min). Myricetin was found at a concentration of approximately 30 pmol in the ocular tissue 30 min after intraperitoneal injection (using HPLC-MS), showing that this substance can cross the BRB. However, quercetin is not easily detected, possibly owing to its detection limit. The protective effects of myricetin and quercetin are probably linked to the upregulation of photoreceptor-specific proteins (rhodopsin and cone opsins) and downregulation of proinflammatory chemokines and cytokines, leading to BL-mediated apoptosis suppression [33]. A daily oral dose of myricetin (25–100 mg/kg b.w./day) for 6 weeks prevented intraocular pressure elevation and reduced oxidative stress in rats with hyaluronic acid-induced glaucoma, according to a different study on myricetin eye protection [78]. In rats, a single intraperitoneal injection of high-dose myricetin (20 mg/kg b.w.) altered circadian rhythms by reducing nocturnal serum melatonin levels and locomotor activity by decreasing acetyl coenzyme A: arylalkylamine *N*-acetyltransferase activity [79].

Laabich et al. [34] found that when primary bovine retinal cells were exposed to BL (420 nm; 500 lux, 17.4 W/m^2^) for 20 h, 75% of the photoreceptors perished. This phototoxicity is remarkedly attenuated in cells pretreated with myricetin (5–40 μM), whereas quercetin and kaempferol have only mild beneficial effects at the same concentrations. The number of hydroxyl groups in their structures may be associated with the antioxidant activity of flavonoids (that is, their ability to scavenge free radicals) [34]. Myricetin has three hydroxyl groups in its B ring and is hypothesized to be more effective than quercetin and kaempferol in response to BL challenge. Furthermore, myricetin and quercetin have greater free radical-scavenging properties than those of kaempferol [80]. Additionally, A2E causes dose-dependent primary bovine photoreceptor and bipolar cell apoptosis. In the absence of BL, treatment with myricetin, quercetin, and kaempferol protects cells from A2E toxicity.

#### 3.1.5. Cynaroside

Cynaroside, also known as luteolin 7-glucoside or luteoloside, is a glycosyloxyflavone of luteolin found in numerous fruits and cruciferous vegetables, such as berries, lemons, oranges, grapes, red cabbage, kale, spinach, lettuce, and carrots. According to the scientific literature, cynaroside exhibits antioxidant and antibacterial properties [81]. In the A2E-laden ARPE-19 cell model, pretreatment with 10 and 20 μM of cynaroside inhibits NF-κB signaling and NLRP3 inflammasome activation, normalizes oxidative stress-related markers, and reduces apoptotic cell death caused by BL (430 nm, 2500 lux for 2 h) [35]. Cynaroside also prevents H_2_O_2_-induced cytotoxicity in ARPE-19 cells [82]. Similar antiapoptotic and antioxidative effects were observed in rats administered 5 μg cynaroside intravitreally while exposed to high-intensity BL irradiation (10,000 lux, 2 h/day) for 7 d.

#### 3.1.6. Procyanidin B2

One of the principal proanthocyanidins found in grape seeds is procyanidin B2, a B-type proanthocyanidin consisting of two (−)-epicatechin molecules [83]. Presently, there are no in vivo studies on procyanidin B2 focusing on photodamage, and data on the distribution of procyanidins in the retina are unavailable. However, Yamakoshi et al. [84] reported that procyanidin-rich grape seed extracts exhibited anticataract activity in hereditary cataractous rats. The antiglycation properties of procyanidin B2 prevented the onset of cataracts and retinopathy in rats with streptozotocin-induced diabetes. Procyanidin-B2 reduced the expression of GFAP and vascular endothelial growth factor (VEGF) in the diabetic retina [85]. These findings suggest that procyanidins or their active metabolites can cross the BRB and enter ocular tissues.

In an A2E-laden ARPE-19 cell model, treatment with procyanidin B2 [36] or grape skin extracts containing resveratrol, catechin, epicatechin, anthocyanins, quercetin, oligomeric procyanidins, and phenolic acids [40] prevented BL-induced ROS generation, ER stress, and mitochondria-dependent apoptosis. Mice fed a grape-enriched diet showed improved photoreceptor survival and ERG-based functions in a model of retinal degeneration induced by subretinal paraquat injection [86]. Among the 19 natural compounds isolated from ginkgo biloba extracts, procyanidin B2 is particularly effective in preventing tert-butyl hydroperoxide-induced oxidative stress and ARPE-19 cell death at a concentration of 10 μM [87]. Sea buckthorn (*Hippophae rhamnoides* L.) extracts, which contain 39% proanthocyanidins, protected the retina of pigmented rabbits from extremely high-intensity visible light (18,000 lux)-induced photoreceptor lesions and suppressed lipid peroxidation and proinflammatory cytokine levels [88]. Without being conjugated or methylated, procyanidin B2 is only marginally absorbed [89]. As a result, the health effects of procyanidin B2 may be attributed, at least in part, to its metabolites rather than to procyanidin B2 itself. Spencer et al. [90] determined that (−)-epicatechin monomer is the predominant metabolite of procyanidin B2 in an isolated procyanidin B2-perfused small intestine. Furthermore, 53 metabolites exhibiting methylation, sulfation, hydration, hydroxylation, hydrogenation, and glucuronidation were detected using UPLC-DAD-ESI-IT-TOF in mice administered pure procyanidin B2 [91].

#### 3.1.7. Phloroglucinol

Phloroglucinol is abundant in the edible alga Ecklonia cava. Piao et al. [92] demonstrated that phloroglucinol protects human HaCaT keratinocytes from UVB radiation by modulating MAPK-AP-1-MMP-1 signaling. Furthermore, Cia et al. [93] reported that phloroglucinol protects primary cultures of RPE and photoreceptor cells against all-trans-retinal-induced ROS formation and cytotoxicity. In cotreatment culture media, ~60% of all-trans-retinal can be trapped by phloroglucinol to produce a stable chromene adduct in an equimolar ratio, as demonstrated by 1H, 13C NMR spectra, and mass analysis. Additionally, phloroglucinol inhibits A2E synthesis in vitro by competing with ethanolamine and all-trans-retinal for binding to the retinal carbonyl group. Because phloroglucinol has low bioavailability and poor lipid solubility, it remains to be determined whether the concentration used in these tests (50 μg/mL) can be detected in a physiologically relevant context. Taveau et al. [94] synthesized a phloroglucinol-based lipophenol derivative, isopropyl-phloroglucinol-DHA, to investigate its protective efficacy against acute photodamage in albino *Abca4*^−/−^ mice. They found that intravenous injection of isopropyl-phloroglucinol-DHA (5–30 mg/kg b.w.) inhibited photoreceptor degradation and ERG-based reduction of a- and b-wave amplitudes caused by 470 nm cool-white fluorescent lamps. These photoprotective effects persisted for 3 months following a light insult and are comparable to the beneficial effects of emixustat, a therapeutic drug used for treating Stargardt disease [95].

#### 3.1.8. Silibinin

Silibinin, also known as silybin, is the primary bioactive flavonoid compound found in 60–70% of *Silybum marianum* L. (milk thistle) varieties. Silibinin is composed of two diastereomers, silybin A and silybin B, in an approximately equimolar ratio, and has been reported to exhibit multiple health benefits such as antioxidant, anti-inflammatory, hepatoprotective, neuroprotective, and antiviral activities [96]. Under hypoxic conditions, treating immortalized RPE cells with silibinin inhibited VEGF secretion by modulating the PI3K-Akt-mTOR-p70S6K signaling pathway [97]. Silibinin protects retinal ganglion cells against BL-induced inflammatory responses and apoptosis via the MEK-ERK-CREB pathway [37]. According to Tvrdy et al. [98], the oral bioavailability of silymarin flavonolignans in humans and rats ranges from 0.45% to 5%, likely owing to their poor aqueous solubility. Nevertheless, in vitro biological effects observed at silibinin concentrations greater than 20–30 μM have little therapeutic relevance because this flavonoid’s maximal achievable plasma concentration is around 10 μM [96].

### 3.2. Lipoic Acid

Lipoic acid is an amphiphilic molecule and metabolic enzyme cofactor with potent antioxidant, anti-inflammatory, and metal-chelating properties. According to clinical investigations, a single oral dose of 2400 mg or daily doses of 1800 mg of lipoic acid for 6 months had no adverse effects on individuals [99]. Because lipoic acid can cross the blood–brain barrier, it may exhibit neuroprotective effects in the central nervous system and eyes [100]. Supplementation with lipoic acid suppressed malondialdehyde (MDA) production, enhanced antioxidant enzyme activity, and lowered corneal and scleral damage induced by UVA [101] and UVB [102] in rabbits and mice. In streptozocin-induced diabetic rats, lipoic acid treatment normalized intraretinal manganese ion uptake [103] and oxygenation response [104] without losing ONL thickness or BRB integrity. By reducing apoptosis, inflammation, oxidative stress, and iron-related gene expression, intraperitoneal injection of lipoic acid at a dose of 100 mg/kg b.w. preserved ERG functions and photoreceptor survival in BALB/c mice with acute light damage (10,000 lux cool-white LED light for 4 h) [105]. These findings further support the hypothesis that lipoic acid-rich extracts, such as those from Ginkgo biloba, red berries, and white willow bark, may have photoprotective potential that requires further investigation.

### 3.3. Prunella vulgaris L. Extracts

*Prunella vulgaris* L. is a widely grown perennial plant that is commonly used as an ingredient in foods, drinks, and traditional medicines. *P. vulgaris* extracts enhance wound healing; alleviate sore throat, fever, and skin allergies; and exert antioxidant, anti-inflammatory, and antiallergic effects; thus, they possess substantial therapeutic potential [106]. According to Kim et al. [41], water extracts of *P. vulgaris* prevent BL-induced apoptosis in A2E-laden ARPE-19 cells and reduce the internalization of A2E. In addition, *P. vulgaris* extracts (50–200 mg/kg bw) activate Nrf2/HO-1 signaling and inhibit NF-κB nuclear translocation in mice exposed to short-term, high-intensity BL. The thickness of ONL and the levels of oxidative markers (ROS, MDA, and GSH), inflammatory cytokines (IL-1, IL-6, and MCP-1), and angiogenesis factors (HIF-1α and VEGF) are improved in *P. vulgaris* extracts-treated groups compared to those in non-treated groups.

## 4. Conclusions

Prolonged exposure to screens can lead to visual impairments, as the chronic phototoxic effects of repeated blue light exposure contribute to the deterioration of retinal functions and morphological integrity. Given the pivotal role of photooxidative stress in the pathogenesis of retinal disorders, the consumption of antioxidant-rich foods such as leafy green vegetables, berries, and nuts, or adherence to healthy dietary patterns like the Mediterranean diet, which includes a variety of fruits, vegetables, and whole grains, may offer a strategic approach to mitigating the detrimental effects of BL exposure. This perspective emphasizes identifying naturally occurring phytochemicals with potential photoprotective properties as a promising avenue for reducing retinal photodamage. Furthermore, actionable recommendations for future research should be considered, such as the need for randomized controlled trials, standardized phytochemical dosages, and exploring synergistic effects between dietary components. These efforts will help advance our understanding of how diet and phytochemicals can protect retinal health from the harmful effects of prolonged blue light exposure.

## Figures and Tables

**Table 1:** Protective effects of polyphenolic compounds against blue and white LED light-induced retinal photodamage in vitro and in vivo.

Compounds	Phototoxic Lighting	Experimental Models	Effects	References
Resveratrol	A2E (10 or 20 μM) + BL (430 nm, 4.02 J/cm^2^, 7 min for 3 day)	ARPE-19 cells	↓ Oxidized A2E formation and intracellular A2E accumulation	[26]
Curcumin	Cool-white LED (1000 lux, 3 h, 09:00–12:00)	Wistar rats	Inhibits NF-κB activation and downregulates the inflammatory gene expression	[27]
	A2E (20 μM) + BL (450 nm, 9.4 mW/cm^2^)	ARPE-19 cells	Inhibits c-Abl and p53 mRNA expression to prevent apoptosis	[28]
Cyanidin-3-glucoside	A2E (3 μM) + BL (430 nm, 1.5 mW/cm^2^, 30 min)	ARPE-19 cells	Scavenges ROS and inhibits A2E photooxidation	[29]
Malvidin-3-glucoside	BL for 30 min	ARPE-19 cells	Inhibits mitochondrial damage and ROS production	[30]
Quercetin	White fluorescent light (3000 lux)	Sprague–Dawley rats	↓ 8-OHdG level and TUNEL-positive cells	[31]
	A2E (3 μM) + BL (430 nm, 1.5 mW/cm^2^, 30 min)	ARPE-19 cells	Inhibits proinflammatory cytokine release and cell apoptosis Inhibits MG-H1 levels and RAGE mRNA expression	[29]
Quercetin-3-*O*-α-l-arabinopyranoside	Blue–green light (480 nm, 4000 lux, 10 min)	ARPE-19 cells Balb-c mice	Inhibits NF-κB, AP-1, and C3 expression Inhibits the pyrolysis of poly polymerases Inhibits the thinning of the INL, OPL, and ONL in the mouse retina	[32]
Myricetin and quercetin	Bright light (10,000 lux, 45 min)	*Abca4*^−/−^*Rdh8*^−/−^ mice	Inhibits inflammatory responses and photoreceptor apoptosis	[33]
Myricetin	BL (420 nm, 500 lux, 17.4 W/m^2^, 20 h); A2E (30 μM)	Primary bovine retinal cells	Prevents BL-induced and A2E-induced death of primary photoreceptor cells in the retina	[34]
Cynaroside	A2E (25 μM) + BL (430 nm, 2500 lux, 2 h)	ARPE-19 cells	Impedes NF-κB signaling and NLRP3 inflammasome activation Normalizes oxidative stress-related markers Inhibits apoptotic cell death	[35]
Procyanidin B2	A2E (25 μM) + BL (2000 lux, 30 min)	ARPE-19 cells	Prevents ROS generation, ER stress, and mitochondria-dependent apoptosis	[36]
Silibinin	BL (530 nm with a peak of 470 nm, 12.08 W/m^2^, 24 h)	Retinal ganglion cells	Inhibits apoptosis	[37]

The symbol “↓” means decrease. A2E, *N*-retinylidene-*N*-retinylethanolamine; BL, blue light; ARPE, adult retinal pigment epithelial; LED, light-emitting diodes; NF-κB, nuclear factor kappa-light-chain-enhancer of activated B cells; ROS, reactive oxygen species; 8-OHdG, 8-hydroxy-2-deoxyguanosine; TUNEL, terminal deoxynucleotidyl transferase dUTP nick end labeling; MG-H1, methylglyoxal-derived hydroimidazolones; RAGE, receptor for advanced glycation end products; AP-1, activator protein 1; INL, inner nuclear layer; OPL, outer plexiform layer; ONL, outer nuclear layer.

**Table 2:** Protective effects of plant extracts against blue LED light-induced retinal photodamage in vitro and in vivo.

Plant Extracts	Phototoxic Lighting	Experimental Models	Effects	References
Blueberry anthocyanin-rich extracts	White LED (420–800 nm, 2500 lux for 12 h)	ARPE-19 cells	Inhibits cellular senescence	[38]
*Vaccinium myrtillus* L. (bilberry) and *Vaccinium vitis-idaea* (lingonberry)	BL (460–470 nm, 2500 lux, 6 h)	Photoreceptor 661W cells	Inhibits the generation of ROS and regulates the activation of NF-κB, p38, MAPK, and caspase-3/7 to protect retinal photoreceptor cells	[39]
Grape skin	BL (2000 lux, 30 min)	ARPE-19 cells	Inhibits the ER stress-mediated intrinsic apoptotic pathway	[40]
*Prunella vulgaris* var. L	A2E (20 μM) + BL (430 nm, 4000 lux, 10 min)	ARPE-19 cells	Activates Nrf2/HO-1 signaling, inhibits ROS and MDA production ↓ inflammation	[41]
	BL (430 nm, 10,000 lux, 1 h/day for 14 d)	BALB/c mice		

The symbol “↓” means decrease. BL, blue light; ROS, reactive oxygen species; NF-κB, nuclear factor kappa-light-chain-enhancer of activated B cells; MAPK, mitogen-activated protein kinase; ARPE, adult retinal pigment epithelial; Bax, Bcl-2 associated X; Bcal-2, B-cell lymphoma 2; FasL, Fas ligand; TUNEL, terminal deoxynucleotidyl transferase dUTP nick end labeling; ONL, outer nuclear layer; ER, endoplasmic reticulum; A2E, *N*-retinylidene-*N*-retinylethanolamine; Nrf2, NFE2-related factor 2; HO-1, heme oxygenase-1; MDA, malondialdehyde.

## References

[1] Cougnard-Gregoire A., Merle B.M.J., Aslam T., Seddon J.M., Aknin I., Klaver C.C.W., Garhöfer G., Layana A.G., Minnella A.M., Silva R. (2023). Blue Light Exposure: Ocular Hazards and Prevention—A Narrative Review. Ophthalmol. Ther..

[2] Behar-Cohen F., Martinsons C., Viénot F., Zissis G., Barlier-Salsi A., Cesarini J.P., Enouf O., Garcia M., Picaud S., Attia D. (2011). Light-emitting diodes (LED) for domestic lighting: Any risks for the eye?. Prog. Retin. Eye Res..

[3] Wong N.A., Bahmani H. (2022). A review of the current state of research on artificial blue light safety as it applies to digital devices. Heliyon.

[4] Yeh W.-J., Chien P.-T., Wen Y.-T., Wu C.-H. (2024). A comprehensive review of experimental models for investigating blue light-induced ocular damage: Insights into parameters, limitations, and new opportunities. Exp. Eye Res..

[5] Ratnayake K., Payton J.L., Meger M.E., Godage N.H., Gionfriddo E., Karunarathne A. (2020). Blue light-triggered photochemistry and cytotoxicity of retinal. Cell Signal.

[6] Marie M., Bigot K., Angebault C., Barrau C., Gondouin P., Pagan D., Fouquet S., Villette T., Sahel J.A., Lenaers G. (2018). Light action spectrum on oxidative stress and mitochondrial damage in A2E-loaded retinal pigment epithelium cells. Cell Death Dis..

[7] Zhang Z.Y., Bao X.L., Cong Y.Y., Fan B., Li G.Y. (2020). Autophagy in Age-Related macular degeneration: A regulatory mechanism of oxidative stress. Oxidative Med. Cell Longev..

[8] Datta S., Cano M., Ebrahimi K., Wang L., Handa J.T. (2017). The impact of oxidative stress and inflammation on RPE degeneration in non-neovascular AMD. Prog. Retin. Eye Res..

[9] Kagan V.E., Shvedova A.A., Novikov K.N., Kozlov Y.P. (1973). Light-induced free radical oxidation of membrane lipids in photoreceptors of frog retina. Biochim. Biophys. Acta (BBA)-Biomembr..

[10] Hunter J.J., Morgan J.I., Merigan W.H., Sliney D.H., Sparrow J.R., Williams D.R. (2012). The susceptibility of the retina to photochemical damage from visible light. Prog. Retin. Eye Res..

[11] Kim G.H., Kim H.I., Paik S.S., Jung S.W., Kang S., Kim I.B. (2016). Functional and morphological evaluation of blue light-emitting diode-induced retinal degeneration in mice. Graefes Arch. Clin. Exp. Ophthalmol..

[12] Contin M.A., Arietti M.M., Benedetto M.M., Bussi C., Guido M.E. (2013). Photoreceptor damage induced by low-intensity light: Model of retinal degeneration in mammals. Mol. Vis..

[13] Putting B.J., Van Best J.A., Vrensen G.F., Oosterhuis J.A. (1994). Blue-light-induced dysfunction of the blood-retinal barrier at the pigment epithelium in albino versus pigmented rabbits. Exp. Eye Res..

[14] Putting B.J., Zweypfenning R.C., Vrensen G.F., Oosterhuis J.A., van Best J.A. (1992). Blood-retinal barrier dysfunction at the pigment epithelium induced by blue light. Investig. Ophthalmol. Vis. Sci..

[15] Park Y.S., Kim H.L., Lee S.H., Zhang Y., Kim I.B. (2021). Expression of the endoplasmic reticulum stress marker GRP78 in the normal retina and retinal degeneration induced by blue LED stimuli in mice. Cells.

[16] Kuse Y., Ogawa K., Tsuruma K., Shimazawa M., Hara H. (2014). Damage of photoreceptor-derived cells in culture induced by light emitting diode-derived blue light. Sci. Rep..

[17] Ouyang X., Yang J., Hong Z., Wu Y., Xie Y., Wang G. (2020). Mechanisms of blue light-induced eye hazard and protective measures: A review. Biomed. Pharmacother..

[18] Cabrera M.P., Chihuailaf R.H. (2011). Antioxidants and the integrity of ocular tissues. Vet. Med. Int..

[19] Bungau S., Abdel-Daim M.M., Tit D.M., Ghanem E., Sato S., Maruyama-Inoue M., Yamane S., Kadonosono K. (2019). Health Benefits of polyphenols andcarotenoids in age-related eye diseases. Oxidative Med. Cell Longev..

[20] The Age-Related Eye Disease Study Research Group (1999). The Age-Related Eye Disease Study (AREDS): Design implications. AREDS report no. 1. Control Clin. Trials.

[21] Group A.R., Chew E.Y., Clemons T., SanGiovanni J.P., Danis R., Domalpally A., McBee W., Sperduto R., Ferris F.L. (2012). The Age-Related Eye Disease Study 2 (AREDS2): Study design and baseline characteristics (AREDS2 report number 1). Ophthalmology.

[22] D’Angelo A., Vitiello L., Gagliardi V., Salerno G., De Pascale I., Coppola A., Abbinante G., Pellegrino A., Giannaccare G. (2024). The role of oral supplementation for the management of age-related macular degeneration: A narrative review. J. Pers. Med..

[23] Bernstein P.S., Li B., Vachali P.P., Gorusupudi A., Shyam R., Henriksen B.S., Nolan J.M. (2016). Lutein, zeaxanthin, and meso-zeaxanthin: The basic and clinical science underlying carotenoid-based nutritional interventions against ocular disease. Prog. Retin. Eye Res..

[24] Yang J., Li D., Zhang Y., Zhang L., Liao Z., Aihemaitijiang S., Hou Y., Zhan Z., Xie K., Zhang Z. (2020). Lutein protected the retina from light induced retinal damage by inhibiting increasing oxidative stress and inflammation. J. Funct. Foods.

[25] Choo P.P., Woi P.J., Bastion M.C., Omar R., Mustapha M., Md Din N. (2022). Review of evidence for the usage of antioxidants for eye aging. Biomed. Res. Int..

[26] Kang J.H., Choung S.Y. (2016). Protective effects of resveratrol and its analogs on age-related macular degeneration in vitro. Arch. Pharm. Res..

[27] Mandal M.N., Patlolla J.M., Zheng L., Agbaga M.P., Tran J.T., Wicker L., Kasus-Jacobi A., Elliott M.H., Rao C.V., Anderson R.E. (2009). Curcumin protects retinal cells from light-and oxidant stress-induced cell death. Free Radic. Biol. Med..

[28] Park S.I., Lee E.H., Kim S.R., Jang Y.P. (2017). Anti-apoptotic effects of Curcuma longa L. extract and its curcuminoids against blue light-induced cytotoxicity in A2E-laden human retinal pigment epithelial cells. J. Pharm. Pharmacol..

[29] Wang Y., Kim H.J., Sparrow J.R. (2017). Quercetin and cyanidin-3-glucoside protect against photooxidation and photodegradation of A2E in retinal pigment epithelial cells. Exp. Eye Res..

[30] Yacout S.M., Gaillard E.R. (2017). The Anthocyanins, oenin and callistephin, protect RPE cells against oxidative stress. Photochem. Photobiol..

[31] Koyama Y., Kaidzu S., Kim Y.C., Matsuoka Y., Ishihara T., Ohira A., Tanito M. (2019). Suppression of Light-Induced Retinal Degeneration by Quercetin via the AP-1 Pathway in Rats. Antioxidants.

[32] Kim J., Jin H.L., Jang D.S., Jeong K.W., Choung S.-Y. (2018). Quercetin-3-*O*-α-l-arabinopyranoside protects against retinal cell death via blue light-induced damage in human RPE cells and Balb-c mice. Food Funct..

[33] Ortega J.T., Parmar T., Golczak M., Jastrzebska B. (2021). Protective Effects of flavonoids in acute models of light-induced retinal degeneration. Mol. Pharmacol..

[34] Laabich A., Manmoto C.C., Kuksa V., Leung D.W., Vissvesvaran G.P., Karliga I., Kamat M., Scott I.L., Fawzi A., Kubota R. (2007). Protective effects of myricetin and related flavonols against A2E and light mediated-cell death in bovine retinal primary cell culture. Exp. Eye Res..

[35] Feng J.H., Dong X.W., Yu H.L., Shen W., Lv X.Y., Wang R., Cheng X.X., Xiong F., Hu X.L., Wang H. (2021). Cynaroside protects the blue light-induced retinal degeneration through alleviating apoptosis and inducing autophagy in vitro and in vivo. Phytomedicine.

[36] Li W., Jiang Y., Sun T., Yao X., Sun X. (2016). Supplementation of procyanidins B2 attenuates photooxidation-induced apoptosis in ARPE-19 cells. Int. J. Food Sci. Nutr..

[37] Shen Y., Zhao H., Wang Z., Guan W., Kang X., Tai X., Sun Y. (2019). Silibinin declines blue light-induced apoptosis and inflammation through MEK/ERK/CREB of retinal ganglion cells. Artif. Cells Nanomed. Biotechnol..

[38] Liu Y., Song X., Zhang D., Zhou F., Wang D., Wei Y., Gao F., Xie L., Jia G., Wu W. (2012). Blueberry anthocyanins: Protection against ageing and light-induced damage in retinal pigment epithelial cells. Br. J. Nutr..

[39] Ogawa K., Kuse Y., Tsuruma K., Kobayashi S., Shimazawa M., Hara H. (2014). Protective effects of bilberry and lingonberry extracts against blue light-emitting diode light-induced retinal photoreceptor cell damage in vitro. BMC Complement. Altern. Med..

[40] Zhao Z., Sun T., Jiang Y., Wu L., Cai X., Sun X., Sun X. (2014). Photooxidative damage in retinal pigment epithelial cells via GRP78 and the protective role of grape skin polyphenols. Food Chem. Toxicol..

[41] Kim J., Cho K., Choung S.Y. (2020). Protective effect of *Prunella vulgaris* var. L extract against blue light induced damages in ARPE-19 cells and mouse retina. Free Radic. Biol. Med..

[42] Pintea A., Rugină D. (2019). Resveratrol and the human retina. Handbook of Nutrition, Diet, and the Eye.

[43] Alarcon De La Lastra C., Villegas I. (2005). Resveratrol as an anti-inflammatory and anti-aging agent: Mechanisms and clinical implications. Mol. Nutr. Food Res..

[44] Walle T. (2011). Bioavailability of resveratrol. Ann. N. Y. Acad. Sci..

[45] Chan C.M., Huang C.H., Li H.J., Hsiao C.Y., Su C.C., Lee P.L., Hung C.F. (2015). Protective effects of resveratrol against UVA-induced damage in ARPE19 cells. Int. J. Mol. Sci..

[46] Liu Y., Chan F., Sun H., Yan J., Fan D., Zhao D., An J., Zhou D. (2011). Resveratrol protects human keratinocytes HaCaT cells from UVA-induced oxidative stress damage by downregulating Keap1 expression. Eur. J. Pharmacol..

[47] Pinelli R., Bertelli M., Scaffidi E., Bumah V.V., Biagioni F., Busceti C.L., Puglisi-Allegra S., Fornai F. (2021). The neurobiology of nutraceuticals combined with light exposure, a case report in the course of retinal degeneration. Arch. Ital. Biol..

[48] Richer S., Stiles W., Ulanski L., Carroll D., Podella C. (2013). Observation of human retinal remodeling in octogenarians with a resveratrol based nutritional supplement. Nutrients.

[49] Hu W.H., Zhang X.Y., Leung K.W., Duan R., Dong T.T., Qin Q.W., Tsim K.W. (2022). Resveratrol, an inhibitor binding to VEGF, restores the pathology of abnormal angiogenesis in retinopathy of prematurity (ROP) in mice: Application by intravitreal and topical instillation. Int. J. Mol. Sci..

[50] Shakibaei M., Harikumar K.B., Aggarwal B.B. (2009). Resveratrol addiction: To die or not to die. Mol. Nutr. Food Res..

[51] Dos Santos D.P., Soares Lopes D.P., de Moraes R.C.J., Vieira Goncalves C., Pereira Rosa L., da Silva Rosa F.C., da Silva R.A.A. (2019). Photoactivated resveratrol against *Staphylococcus aureus* infection in mice. Photodiagnosis Photodyn. Ther..

[52] Souza E.Q.M., da Rocha T.E., Toro L.F., Guiati I.Z., Freire J.d.O.A., Ervolino E., Brandini D.A., Garcia V.G., Theodoro L.H. (2021). Adjuvant effects of curcumin as a photoantimicrobial or irrigant in the non-surgical treatment of periodontitis: Systematic review and meta-analysis. Photodiagnosis Photodyn. Ther..

[53] Muniz I.P.R., Galantini M.P.L., Ribeiro I.S., Goncalves C.V., Dos Santos D.P., Moura T.C., Silva E.S., Silva N.R., Cipriano B.P., Correia T.M.L. (2021). Antimicrobial photodynamic therapy (aPDT) with curcumin controls intradermal infection by *Staphylococcus aureus* in mice with type 1 diabetes mellitus: A pilot study. J. Photochem. Photobiol. B.

[54] Cossu M., Ledda L., Cossu A. (2021). Emerging trends in the photodynamic inactivation (PDI) applied to the food decontamination. Food Res. Int..

[55] Polat E., Kang K. (2021). Natural Photosensitizers in Antimicrobial Photodynamic Therapy. Biomedicines.

[56] Dahl T.A., Bilski P., Reszka K.J., Chignell C.F. (1994). Photocytotoxicity of curcumin. Photochem. Photobiol..

[57] Banerjee S., Prasad P., Hussain A., Khan I., Kondaiah P., Chakravarty A.R. (2012). Remarkable photocytotoxicity of curcumin in HeLa cells in visible light and arresting its degradation on oxovanadium(IV) complex formation. Chem. Commun..

[58] Seidi Damyeh M., Mereddy R., Netzel M.E., Sultanbawa Y. (2020). An insight into curcumin-based photosensitization as a promising and green food preservation technology. Compr. Rev. Food Sci. Food Saf..

[59] Anand P., Kunnumakkara A.B., Newman R.A., Aggarwal B.B. (2007). Bioavailability of curcumin: Problems and promises. Mol. Pharm..

[60] Noureddin S.A., El-Shishtawy R.M., Al-Footy K.O. (2019). Curcumin analogues and their hybrid molecules as multifunctional drugs. Eur. J. Med. Chem..

[61] Kähkönen M.P., Hopia A.I., Heinonen M. (2001). Berry phenolics and their antioxidant activity. J. Agric. Food Chem..

[62] Huang Q., Braffett B.H., Simmens S.J., Young H.A., Ogden C.L. (2020). Dietary polyphenol intake in US adults and 10-year trends: 2007–2016. J. Acad. Nutr. Diet..

[63] Igwe E.O., Charlton K.E., Probst Y.C. (2019). Usual dietary anthocyanin intake, sources and their association with blood pressure in a representative sample of Australian adults. J. Hum. Nutr. Diet..

[64] Nomi Y., Iwasaki-Kurashige K., Matsumoto H. (2019). Therapeutic effects of anthocyanins for vision and eye health. Molecules.

[65] Wang Y., Huo Y., Zhao L., Lu F., Wang O., Yang X., Ji B., Zhou F. (2016). Cyanidin-3-glucoside and its phenolic acid metabolites attenuate visible light-induced retinal degeneration in vivo via activation of Nrf2/HO-1 pathway and NF-kappaB suppression. Mol. Nutr. Food Res..

[66] Czank C., Cassidy A., Zhang Q., Morrison D.J., Preston T., Kroon P.A., Botting N.P., Kay C.D. (2013). Human metabolism and elimination of the anthocyanin, cyanidin-3-glucoside: A (13)C-tracer study. Am. J. Clin. Nutr..

[67] Jang Y.P., Zhou J., Nakanishi K., Sparrow J.R. (2005). Anthocyanins protect against A2E photooxidation and membrane permeabilization in retinal pigment epithelial cells. Photochem. Photobiol..

[68] Katalinić V., Možina S.S., Skroza D., Generalić I., Abramovič H., Miloš M., Ljubenkov I., Piskernik S., Pezo I., Terpinc P. (2010). Polyphenolic profile, antioxidant properties and antimicrobial activity of grape skin extracts of 14 *Vitis vinifera* varieties grown in Dalmatia (Croatia). Food Chem..

[69] Yu C.C., Nandrot E.F., Dun Y., Finnemann S.C. (2012). Dietary antioxidants prevent age-related retinal pigment epithelium actin damage and blindness in mice lacking alphavbeta5 integrin. Free Radic. Biol. Med..

[70] Kalt W., Blumberg J.B., McDonald J.E., Vinqvist-Tymchuk M.R., Fillmore S.A., Graf B.A., O’Leary J.M., Milbury P.E. (2008). Identification of anthocyanins in the liver, eye, and brain of blueberry-fed pigs. J. Agric. Food Chem..

[71] Matsumoto H., Nakamura Y., Iida H., Ito K., Ohguro H. (2006). Comparative assessment of distribution of blackcurrant anthocyanins in rabbit and rat ocular tissues. Exp. Eye Res..

[72] Zhao L., Wang H., Du X. (2021). The therapeutic use of quercetin in ophthalmology: Recent applications. Biomed. Pharmacother..

[73] Zhou J., Ueda K., Zhao J., Sparrow J.R. (2015). Correlations between photodegradation of bisretinoid constituents of retina and dicarbonyl adduct deposition. J. Biol. Chem..

[74] Cao X., Liu M., Tuo J., Shen D., Chan C.C. (2010). The effects of quercetin in cultured human RPE cells under oxidative stress and in Ccl2/Cx3cr1 double deficient mice. Exp. Eye Res..

[75] Maher P., Hanneken A. (2005). Flavonoids protect retinal ganglion cells from oxidative stress–induced death. Investig. Ophthalmol. Vis. Sci..

[76] Taheri Y., Suleria H.A.R., Martins N., Sytar O., Beyatli A., Yeskaliyeva B., Seitimova G., Salehi B., Semwal P., Painuli S. (2020). Myricetin bioactive effects: Moving from preclinical evidence to potential clinical applications. BMC Complement. Med. Ther..

[77] Ortega J.T., Parmar T., Jastrzebska B. (2019). Flavonoids enhance rod opsin stability, folding, and self-association by directly binding to ligand-free opsin and modulating its conformation. J. Biol. Chem..

[78] Yang Q., Li Y., Luo L. (2018). Effect of Myricetin on Primary Open-angle Glaucoma. Transl. Neurosci..

[79] Shin J.C., Jung H.Y., Harikishore A., Kwon O.D., Yoon H.S., Kim K.T., Choi B.H. (2013). The flavonoid myricetin reduces nocturnal melatonin levels in the blood through the inhibition of serotonin N-acetyltransferase. Biochem. Biophys. Res. Commun..

[80] Cao G., Sofic E., Prior R.L. (1997). Antioxidant and prooxidant behavior of flavonoids: Structure-activity relationships. Free Radic. Biol. Med..

[81] Caporali S., De Stefano A., Calabrese C., Giovannelli A., Pieri M., Savini I., Tesauro M., Bernardini S., Minieri M., Terrinoni A. (2022). Anti-inflammatory and active biological properties of the plant-derived bioactive compounds luteolin and luteolin 7-glucoside. Nutrients.

[82] Yu H., Li J., Hu X., Feng J., Wang H., Xiong F. (2019). Protective effects of cynaroside on oxidative stress in retinal pigment epithelial cells. J. Biochem. Mol. Toxicol..

[83] Rodriguez-Ramiro I., Ramos S., Bravo L., Goya L., Martin M.A. (2012). Procyanidin B2 induces Nrf2 translocation and glutathione S-transferase P1 expression via ERKs and p38-MAPK pathways and protect human colonic cells against oxidative stress. Eur. J. Nutr..

[84] Yamakoshi J., Saito M., Kataoka S., Tokutake S. (2002). Procyanidin-rich extract from grape seeds prevents cataract formation in hereditary cataractous (ICR/f) rats. J. Agric. Food Chem..

[85] Reddy G.B., Muthenna P., Akileshwari C., Raghu G., Suryanarayana P. (2013). Antiglycating potential of procyanidin-B2 isolated from cinnamon bark: Prevention or treatment of diabetic ocular complications (cataract & retinopathy). Investig. Ophthalmol. Vis. Sci..

[86] Patel A.K., Davis A., Rodriguez M.E., Agron S., Hackam A.S. (2016). Protective effects of a grape-supplemented diet in a mouse model of retinal degeneration. Nutrition.

[87] Li Y., Cheng Z., Wang K., Zhu X., Ali Y., Shu W., Bao X., Zhu L., Fan X., Murray M. (2021). Procyanidin B2 and rutin in Ginkgo biloba extracts protect human retinal pigment epithelial (RPE) cells from oxidative stress by modulating Nrf2 and Erk1/2 signalling. Exp. Eye Res..

[88] Wang Y., Zhao L., Huo Y., Zhou F., Wu W., Lu F., Yang X., Guo X., Chen P., Deng Q. (2016). Protective effect of proanthocyanidins from sea buckthorn (*Hippophae rhamnoides* L.) seed against visible light-induced retinal degeneration in vivo. Nutrients.

[89] Stoupi S., Williamson G., Viton F., Barron D., King L.J., Brown J.E., Clifford M.N. (2010). In vivo bioavailability, absorption, excretion, and pharmacokinetics of [^14^C] procyanidin B2 in male rats. Drug Metab. Dispos..

[90] Spencer J.P., Schroeter H., Shenoy B., Srai S.K., Debnam E.S., Rice-Evans C. (2001). Epicatechin is the primary bioavailable form of the procyanidin dimers B2 and B5 after transfer across the small intestine. Biochem. Biophys. Res. Commun..

[91] Xiao Y., Hu Z., Yin Z., Zhou Y., Liu T., Zhou X., Chang D. (2017). Profiling and distribution of metabolites of procyanidin B2 in mice by UPLC-DAD-ESI-IT-TOF-MSn technique. Front. Pharmacol..

[92] Piao M.J., Zhang R., Lee N.H., Hyun J.W. (2012). Phloroglucinol attenuates ultraviolet B radiation-induced matrix metalloproteinase-1 production in human keratinocytes via inhibitory actions against mitogen-activated protein kinases and activator protein-1. Photochem. Photobiol..

[93] Cia D., Cubizolle A., Crauste C., Jacquemot N., Guillou L., Vigor C., Angebault C., Hamel C.P., Vercauteren J., Brabet P. (2016). Phloroglucinol protects retinal pigment epithelium and photoreceptor against all-trans-retinal-induced toxicity and inhibits A2E formation. J. Cell Mol. Med..

[94] Taveau N., Cubizolle A., Guillou L., Pinquier N., Moine E., Cia D., Kalatzis V., Vercauteren J., Durand T., Crauste C. (2020). Preclinical pharmacology of a lipophenol in a mouse model of light-induced retinopathy. Exp. Mol. Med..

[95] Zhang J., Kiser P.D., Badiee M., Palczewska G., Dong Z., Golczak M., Tochtrop G.P., Palczewski K. (2015). Molecular pharmacodynamics of emixustat in protection against retinal degeneration. J. Clin. Investig..

[96] Kren V., Valentova K. (2022). Silybin and its congeners: From traditional medicine to molecular effects. Nat. Prod. Rep..

[97] Lin C.H., Li C.H., Liao P.L., Tse L.S., Huang W.K., Cheng H.W., Cheng Y.W. (2013). Silibinin inhibits VEGF secretion and age-related macular degeneration in a hypoxia-dependent manner through the PI-3 kinase/Akt/mTOR pathway. Br. J. Pharmacol..

[98] Tvrdy V., Pourova J., Jirkovsky E., Kren V., Valentova K., Mladenka P. (2021). Systematic review of pharmacokinetics and potential pharmacokinetic interactions of flavonolignans from silymarin. Med. Res. Rev..

[99] Theodosis-Nobelos P., Papagiouvannis G., Tziona P., Rekka E.A. (2021). Lipoic acid. Kinetics and pluripotent biological properties and derivatives. Mol. Biol. Rep..

[100] Gilgun-Sherki Y., Melamed E., Offen D. (2001). Oxidative stress induced-neurodegenerative diseases: The need for antioxidants that penetrate the blood brain barrier. Neuropharmacology.

[101] Demir U., Demir T., Ilhan N. (2005). The protective effect of alpha-lipoic acid against oxidative damage in rabbit conjunctiva and cornea exposed to ultraviolet radiation. Ophthalmologica.

[102] Chen B.Y., Lin D.P., Chang L.S., Huang T.P., Liu H.J., Luk C.P., Lo Y.L., Chang H.H. (2013). Dietary alpha-lipoic acid prevents UVB-induced corneal and conjunctival degeneration through multiple effects. Investig. Ophthalmol. Vis. Sci..

[103] Berkowitz B.A., Roberts R., Stemmler A., Luan H., Gradianu M. (2007). Impaired apparent ion demand in experimental diabetic retinopathy: Correction by lipoic Acid. Investig. Ophthalmol. Vis. Sci..

[104] Roberts R., Luan H., Berkowitz B.A. (2006). alpha-lipoic acid corrects late-phase supernormal retinal oxygenation response in experimental diabetic retinopathy. Investig. Ophthalmol. Vis. Sci..

[105] Zhao L., Wang C., Song D., Li Y., Song Y., Su G., Dunaief J.L. (2014). Systemic administration of the antioxidant/iron chelator alpha-lipoic acid protects against light-induced photoreceptor degeneration in the mouse retina. Investig. Ophthalmol. Vis. Sci..

[106] Psotová J., Kolár M., Sousek J., Svagera Z., Vicar J., Ulrichová J. (2003). Biological activities of Prunella vulgaris extract. Phytother. Res..

